# Considerations for minority ethnic young people in multisystemic therapy

**DOI:** 10.1177/1359104520969762

**Published:** 2020-11-06

**Authors:** Aisling Bunting, Simone Fox, Jai Adhyaru, Amaryllis Holland

**Affiliations:** 1 Royal Holloway University of London, Egham, UK; 2 King’s College London, UK; 3 Central and North West London Mental Health NHS Trust, UK; 4 South West London and Saint George’s Mental Health NHS Trust, UK

**Keywords:** Multisystemic therapy, culture, antisocial behaviour, youth, secondgeneration

## Abstract

Research has indicated that multisystemic therapy (MST) is an effective treatment for youth with antisocial behaviours (Painter & Scannapieco, 2009). This qualitative study explored minority ethnic young peoples’ experiences of MST, focusing on their understanding of their presenting difficulties and aspects of the intervention which facilitated or hindered engagement and change. Seven semi-structured interviews were conducted with London-based young people who had taken part in MST. A constructivist version of grounded theory analysis was employed. Culture-specific theoretical codes emerged; understanding the family culture and the practitioner acting as a cultural broker, consideration of acculturation differences within the family, exploring the young person’s cultural identity and reflecting on cultural differences in the therapeutic relationship. Findings suggest potential advances to MST practice to meet the needs of minority ethnic young people, including the importance of appropriate training and supervision, sensitively working with salient cultural issues such as the impact of acculturation, and consideration of the role of therapist ethnicity and culture.

## Introduction

Multisystemic therapy (MST) is a family- and community-based intervention for young people who exhibit antisocial behaviour (Henggeler & Borduin, 1990). MST was developed in the US and has been extensively researched, implemented globally (Henggeler & Sheidow, 2012) and endorsed as a culturally competent intervention (Brondino et al., 1997). This assertion is based on studies which have included relatively high percentages of minority ethnic families (Butler et al., 2011; Curtis et al., 2004). Nevertheless, randomised control trials that include a subset of non-white families do not equate to valid analysis of ethnic differences in intervention effects (Hohmann & Parron, 1996). Whilst specific factors influencing the implementation of MST have been researched (Kaur et al., 2015; Paradisopoulos et al., 2015; Tighe et al., 2012), only one study to date has explored the influence of cultural factors (Fox et al., 2016), and found that caregivers consider cultural factors important in the engagement and change process in MST. Research with second-generation young people is crucial, as despite decades of attention to the issue, minority ethnic families continue to be less likely to access mental health services than their mainstream counterparts (Wang et al., 2005) and more likely to delay seeking treatment and to drop out (Hoagwood et al., 2010). Bernal and Scharron-del-Rio (2001) suggest that qualitative research with minority ethnic populations could enhance empirically-supported treatments.

### Second-generation young people

Thompson and Crul (2007) define ‘second-generation’ as children born in the host country, of one or more immigrant parents, or those who arrived before primary-school age. Second-generation young people have a unique experience of growing up in dual-cultured families. In terms of parent-child relations, conflicts based on cultural differences are reported (Robila & Sandberg, 2011). To ensure consistency in the context of existing research, the terms ‘minority ethnic’ and ‘second-generation’ are used (Sewell, 2009) with the understanding that these definitions do not capture the full essence of the constructs to which they refer.

### Acculturation

Acculturation refers to the changes which take place following intercultural contact (Berry, 2005). McGoldrick et al. (2005) argue that second-generation adolescents can reject their parents’ ‘ethnic’ values and strive to adapt to the host culture. Intergenerational conflicts often reflect parents and children acculturating differently (Huq et al., 2016) and can lead to adolescent internalisation and externalisation of emotional problems, which can result in low family cohesion (Tseng & Fuligni, 2000). Bhui et al. (2005) suggest further exploration of acculturation and adolescent mental health is necessary given that this is a vulnerable period of identity change.

### Second-generation risk factors

Bywaters et al. (2017) highlight the over-representation of children from some minority ethnic groups within state governed systems. For example, higher numbers subject to state child protection interventions; in the UK 9% of looked after children were of mixed ethnicity and 7% were of Black or Black British ethnicity, despite making up roughly 5% of the overall population (Zayed & Harker, 2015). In the justice system, Bersani (2014) highlights higher rates of offending in second-generation compared to first-generation immigrants. In 2018, 27% of the 10 to 18-year-olds in the UK who received a youth caution or sentence were from a minority ethnic background, compared with 14% in 2010 (Great Britain. Ministry of Justice, youth justice statistics, 2019, p. 18). de Ruiter and Kaser-Boyd (2015) highlighted over-representation of second-generation adolescents within the criminal justice system, and highlighted ‘perceived discrimination’ as being associated with increased externalising behaviours. Bersani’s (2014) arguments for these differences include; conflicting family and social expectations, growing up in resource-deprived contexts with exposure to risk factors, biases within the justice system including racism, or that second-generation merely parallel native-born or ‘majority group’ youth offending rates.

Additionally, there may be a difference in risk-taking behaviours in second-generation youth, including sexual behaviours (Harris, 1999; Jeltova et al., 2005) and substance misuse (Orford et al., 2004) across difference communities. Nevertheless, Stevens and Vollebergh (2008) highlight that unclear terminology and undefined samples means that research exploring problem behaviours in second-generation young people is of variable quality, warranting further research.

### Rationale for study

Whilst there has been research exploring outcomes for different ethnic groups and qualitative research looking at the experience of MST for minority ethnic caregivers, it has been shown that young people have distinct perspectives to their parents (Paradisopoulos et al., 2015). This study aimed to explore the unique perspectives of second-generation young people, to support MST treatment when working with families of diverse cultural backgrounds.

## Method

### Setting

Minority ethnic families who had received MST and been closed for clinical reasons (drop-out or treatment completed) were identified from the databases of three London MST services. Inclusion of families who had stopped before completion of treatment, was to be inclusive of all MST experiences. Exclusion criteria included young people whose parents were born within the European Union, those who were unable to consent and/or who posed a clinical risk to themselves or others.

### Participants

Twenty-one families were identified; eleven were successfully contacted and seven agreed to take part in the study. Participant demographics are displayed in Table 1.

**Table 1.** Demographic summary of participant sample.

Participant	Gender	Age	Parent country of origin	Parent religion	No. of years in the UK	Parents speak basic English	Language spoken at home	Participant self-reported ethnicity	Participant Religion	Time since MST	Education at time of interview	Living at home	No. of convictions	MST therapist gender/Ethnicity*
Jay	M	17	Samoa/Holland	Pentecostal	16-20	Yes	English	Samoan	Atheist	<6months	Full-time	No (LAC)	1-5	M (WB)
Aisha	F	16	Sudan/Kenya	Muslim	>20	Yes	Arabic English	Kenyan/ Sudanese	Muslim	<3 months	Full-time	Yes	0	M (WB)
Zoya	F	18	Georgia	Jehovah’s Witness	11-15	Yes	Georgian English	Georgian	Christian	<24months	Full-time	Yes	0	F (WB)
Toben	M	18	Nigeria	Christian	>20	Yes	Yoruba English	Black British	Christian	<24months	Part-time	Yes	1-5	F (BB)
Safaa	F	15	Morocco	Muslim	>20	Yes	Arabic English	Moroccan	‘Don’t know’	<3months	Full-time	Yes	0	M (WB)
Tatiana	F	14	Democratic Rep. of Congo	Christian	>20	Yes	Lingala French English	Black British	‘Don’t know’	<3 months	Full-time	Yes	0	M (WB)
Hussein	M	15	Iraq	Christian	16-20	Yes	Arabic English	British	Atheist	<12months	Full-time	Yes	0	F (WB)

### Procedure

Ethical approval was granted by an NHS Research Ethics Committee (REC). Informed consent was obtained before conducting interviews (parental consent obtained if participant was <18 years). Interviews were conducted in participant homes and varied in duration from 40 to 90 minutes. An interview schedule was utilised and subsequently amended based on insights from interviews. In line with grounded theory methodology, the interviews were transcribed verbatim, prior to further interviews.

### Analysis

A constructivist version of grounded theory was employed (Charmaz, 2014) as this method seeks to explore processes, meanings and perceptions based on accounts of lived experiences. This comprised of three levels of analysis: initial coding (sentence-by-sentence summaries), focused coding (analysing the most frequent or significant initial codes) and developing theoretical codes which became tentative analytic concepts. Constant comparisons, memo writing and theoretical sampling were employed to construct abstract theoretical understandings (Charmaz, 2014). Data codes were reviewed independently by the researcher and a research supervisor until convergence was reached. Two fellow researchers reviewed codes and emerging concepts, to ensure interpretations fitted the data. Theoretical sufficiency was adopted (Charmaz, 2014), meaning that by write up the author believed the categories developed explained the data sufficiently. These categories and the emerging theory were verified with a participant, in accordance with qualitative research quality guidelines (Elliott et al., 1999).

## Findings

The analysis generated four culture-specific theoretical codes. Table 2 shows each theoretical code was composed of interacting focused codes, which were made up of interacting initial codes. Extracts from the data are provided to evidence the codes. Pseudonyms were used to protect participants.

**Table 2.** Cultural themes.

Theoretical codes	Focused codes	Properties of the codes
1. Understanding Family Culture	Therapist understanding, respecting and being knowledgeable about culture	• Perception that parents believe that their culture is respected
		• Therapist having knowledge about family’s culture/religion
	Addressing different views on outside help	• Therapist being curious about family cultural beliefs of outside help
		• Help-seeking from extended family or faith-based supports
		• Perception of outside help as more acceptable to young person
2. Considering cultural differences within the family	Role of cultural/acculturation differences on young person’s presenting difficulties	• Young person feeling confused by differences – leading to conflict and inconsistencies
		• Young person feeling judged by parents for deviating from family culture
	Therapist being culturally sensitive and facilitating perspective-taking	• Therapist considering cultural differences within family
		• Therapist opening conversations about dual-culture influencing difficulties
		• Therapist discussing differences between family’s culture and host culture and putting young person’s behaviour in context
	Differences in discipline practices	• Different perspectives on cultural practices in relation to discipline
3. Understanding the young person’s unique cultural values/beliefs	Viewing their identity as a cultural blend	• Young person feeling confused by different cultural influences
		• Young person developing beliefs/values which are a blend of cultural influences
		• Young person developing their own sense of right and wrong
	Therapist exploring identity and sharing with family	• Therapist exploring young person’s cultural identity
		• Therapist taking time to explain this to parents
4. Recognising and reflecting on cultural difference in therapeutic relationship	Therapists addressing own ethnicity and cultural identity	• Young person feeling like ethnicity/culture of therapist had no influence on change
		• Young person thinking a therapist from a similar background would promote change
		• Young person worrying therapists from a similar background would be one-sided
		• Therapist addressing their own culture/religion and any differences with family
		• Discussing potential impact of cultural differences on engagement

### Understanding family culture

Some words used by young people when describing ‘culture’ included; ‘rules’, ‘traditions’, ‘activities’, ‘beliefs’ and ‘religion’. Some participants’ accounts contained frequent references to ‘culture’, for some it just formed the backdrop to their story.

#### Therapist understanding, respecting and being knowledgeable about culture

Some young people valued the MST therapist understanding and being aware of different cultural backgrounds.

Safaa: *“It would help if it were someone from the same culture, or before they came, they would know about the culture, and research.”*

Toben: *“‘Cause she was from a similar background, she wasn’t being biased. . .she was understanding of both my parents and me. . .I think a [White] British person would have understood less.”*

Some young people described that when their parents believed the therapist did not understand their culture, or culture-specific parenting practices were questioned, this impacted on their willingness to consider different perspectives and subsequently their engagement.

Aisha: *“If someone comes from a different culture, that’s judging on mum’s culture, is saying. . .’you’re doing something wrong’. . .she’ll automatically be like ‘no, I’m not.’”*

#### Addressing different views on outside help

Some young people described different cultural attitudes towards help-seeking.

Jay: *“So if you’re blatantly. . .physically disabled, then you’re disabled. But mentally they don’t count that. . .so you’re just naughty [in Samoa]. . .so my dad was still thinking that at the beginning. . .His way of dealing with it. . .wasn’t. . .seek for help. It was more. . .they deal with it.”*

Aisha: *“MST stands for so many things, that my culture doesn’t stand for. The reason there is MST is because people have problems. . .in my culture, you’re not really supposed to reach out to someone if you have a problem. You’re the one who’s supposed to fix that problem.”*

Some young people alluded to a sense of shame associated with outside help and conflicting perspectives to their parents on the role of services.

Tatiana: *“In their eyes having a social worker or therapist, it’s like. . .bad to your family, ‘cause in the Congo. . .it’s like reputation, innit? Family name. . .you got to keep your family name as pride.”*

Aisha: *“I contacted social services. . .I brought them here so we could fix things. But mum thinks. . .I just want to get them in trouble. . .The original reason about hitting me. . .[mum] still doesn’t think. . .it’s wrong. . .but when [MST therapist] is here. . .she’ll say ‘yeah I think it’s wrong’ because she wants services to go away.”*

Some young people described their parents’ solutions for behaviour problems.

Toben: *“What they would have suggested would be. . .prayer, yeah praying.”*

Aisha: *“I think they would do what their cultures do. . .send them back to try and fix them. . .my mum. . .was thinking of sending me to Sudan. . .and I was like ‘you try and do that!’”*

Zoya: *“[grandmother] said ‘send her here. . .she obviously doesn’t like living. . .over there, with you’”*

### Considering cultural differences within the family

#### Role of cultural/acculturation differences on young person’s presenting difficulties

Some participants contextualised their difficulties within a cultural framework, seeing the dual-cultures within the family as the source of the conflict; for example, behavioural expectations.

Safaa: *“My dad believed. . .I should not take school as seriously. My parents obviously wouldn’t understand. . .school is compulsory here. In Morocco, it’s not important for a girl to go to school. . .you’re just supposed to learn the household stuff, and taking care of your husband.”*

Toben: *“In Nigeria. . .there’s no form of disrespect. . ..no arguing back. . .I already had a voice. . .but I didn’t have a voice that was listened to. . .she. . .ensured that I was listened to.”*

Hussein: *“If you’re a child in Iraq you don’t argue back, don’t have a side of the argument. You just have to listen. . .I was not going to church, so that caused arguments.”*

Some participants alluded to cultural/acculturation differences between their parents’ as contributing to conflict at home.

Safaa: *“My dad’s really religious, from his culture. But. . .my mum wouldn’t complain about what I’m wearing, or if I have friends that are boys. . .but my dad wouldn’t allow it. That’s what kept the arguments going. . .there was no agreement.”*

Toben: *“The person most in my family that is very clear to the world that he’s Nigerian is my dad. My mum, because she works. . .I think she’s adapted like an English persona. But my dad very much has still got that Nigerian persona. . .I don’t think my dad likes the fact that my mum understands me, and then this creates arguments.”*

#### Therapist being culturally sensitive and facilitating perspective-taking

Most participants alluded to the MST therapist facilitating perspective-taking between parent and young person.

Zoya: *“Yeah talking about religion was a big part [of MST]. . .me being forced to attend meetings as a child. . .and I used to get so much. . .like, abuse. My dad would hit me. . .I had such anger towards him. But. . .learning things made me think, at the end of the day, they’re my parents. . .they do care about me.”*

Tatiana: *“Certain parts of Congo. . .like, how poor people actually are. . .that can’t afford to go to school. . .obviously my mum will tell me, you have to work hard. . .I think she’s seen everything in a different angle to how we see it.”*

Safaa: *“So MST talked about culture, with your problems inside of that. . .like explain to my parents, ‘yeah ‘cause she’s a girl, she would still have to go to school and get her GCSES’. . .but like in Morocco, it’s not important for a girl in school. . .my parents wouldn’t understand that.”*

One participant described a perceived sense of longevity/rigidity in her parents’ views as preventing possible change.

Aisha: *“The therapist would try to explain to them that, I was brought up here and I don’t know any different. . .to try and sort it out. It didn’t really work. I think. . .that my parents feel. . .if you’re coming in to the family. . .and if someone knows something through, like, so many years. . .you can’t just change it by coming in. . .for a few meetings.”*

Young people spoke about their views on UK culture.

Aisha: *“Here. . .it’s ok not to have like a dad, it’s ok to not have a mum. . .or a sister who’s transgender. . .but back home that would be something crazy. . .I think British culture is very free. . .because the culture itself allows everyone else to do their own culture”.*

Safaa: *“[UK] is just more laid back. . .they don’t have. . .proper rules. . .they give you. . .freedom basically.”*

Differences in discipline practices. A number of participants referenced their parents discipline practices in interviews and differences in their views on these.

Aisha: *“In their country it’s ok to hit your child. ‘Cause I was brought up here. . .I think physically hurting someone is bad. It’s not used to teach someone.”*

### Understanding the young persons’ unique cultural values/beliefs

#### Viewing their identity as a culural blend

Many participants described their cultural identity as a blend of both their parent’s culture/religion and peer culture. Some participants alluded to developing their own sense of right and wrong, but feeling this was not understood by their parents’.

Zoya: *“They’re Jehovah’s Witnesses, so they’re very strict and traditional. . .no sex before marriage. . .so I’m more. . .this generation, like, the way British people are. But I don’t believe in having boyfriend after boyfriend, to me that’s disgusting.”*

Hussein: *“I feel like I do the right thing now, but if it’s not. . .how she was brought up. Then it’s drama. . .but she doesn’t get that stuff is different here [UK].”*

Participants alluded to confusion when encountering values/beliefs which were incompatible/contradictory.

Tatiana: *“People say ‘God created the world’, and then. . .other people saying ‘no it wasn’t God’. . .you don’t know what to follow, science or what everyone’s saying. . .so it’s annoying. When I brought it up to mum, she got angry.”*

Some participants alluded to a sense of isolation amongst their peers, linked with navigating their behaviour between two cultures. Others conveyed a sense of marginalisation/discrimination in wider society.

Safaa: *“I can’t have friends that are boys and I’m not allowed to have boyfriends. . .it’s ok but sometimes I think. . .aw other people, like my friends, are allowed, so I feel like I’m missing out.”*

Aisha: *“The events of my religion, like Eid, Ramadan. . .I actually really take that seriously. . .’Cause now, obviously. . .in London, not many people like Muslim people. . .they always judge them. If someone says, ‘uh I don’t like Muslim people’, I’ll be like, ‘I’m Muslim, so what?’”*

#### Therapist exploring identity and sharing with family

Some participants described not feeling understood during MST and valuing one-to-one sessions.

Aisha: *“If they had tried to get to know me first. . .then got to the problem. . .they would have sorted so many more things out. . .I think if I would understand myself a bit more. . .I wouldn’t have done certain things.”*

Toben: *“I feel like it helped. . .but it didn’t work. People didn’t do anything wrong. . .but for improvements. . .they probably could have had more one-to-one sessions than group [family].”*

### Recognising and reflecting on cultural difference in the therapeutic relationship

#### Therapist own ethnicity and cultural identity being addressed

Some participants described thinking that a therapist from a minority ethnic background may have been better placed to engage their parents.

Aisha: *“I think it’s better when the actual therapist is the same religion or background. Like, if someone from your culture is saying. . .’you’re doing something wrong’. . .you’re like ‘oh, wait you’re from my culture, you must know about it, so I should think about what I’m doing. Maybe I’ll not change it, but at least I’ll think about changing’.”*

Two participants expressed concerns that a therapist from a similar background might privilege their parents’ point of view, or that their parents could manipulate them.

Zoya: *“I think they would have understood more, about why the situation is like that, [laughs] . . .actually, this is what I think. . .my mum would try to play with their head. And she’d be like ‘you know how it is’. . .then use more excuses.”*

Safaa: *“If the therapist is Moroccan and my dad’s Moroccan, they’re probably going to be on the same side. . .But. . .if the person can understand both sides. . .then it would be alright.”*

One expressed feeling neutral about a therapist’s background.

Jay: *“Some families would prefer a black social worker?. . .I’ve just always thought that whoever can help me. . .can help me. . .they might talk differently or. . .have different perspectives but. . .in the end they all have the same. . .training.”*

## Discussion

Similarly, to previous MST research (Fox et al., 2016; Kaur et al. 2015; Paradisopoulos et al. 2015; Tighe et al., 2012), this research highlighted some of the universal similarities across cultures. Nonetheless, theoretical codes unique to second-generation young people did emerge.

### Understanding family culture

Therapists having an understanding of different cultural beliefs/values/practices was advocated in this study. Proponents of cultural competence recommend knowledge of specific cultural groups (Lo & Fung, 2003), but endorse a ‘process’ over a ‘content’ model (Lopez et al., 2002). Rober and De Haene (2013) suggest that the ‘cultural competency’ framework, by highlighting the importance of cultural differences and the therapist’s culture-specific knowledge, may underestimate the shared humanity present in a transcultural encounter. Furthermore, appreciating the importance of culture, does not equate to a clear understanding of whether, when, and how to bring up these issues (Cardemil & Battle, 2003).

This research highlighted the different ideas young people and parents have on solutions to family problems. Whilst some parents were described as prioritising faith and extended-family based approaches, young people appear more comfortable with the external services associated with the host culture (social services, police, therapeutic interventions). Higher levels of ‘cultural mistrust’ have been linked to negative attitudes around seeking help from services with majority White staff (Nickerson et al., 1994). Cardemil and Battle (2003) suggest that openness to external help from a therapist of the host culture, may be associated with various identity and acculturation factors. Bhugra and De Silva (2000) advocate discussion around cultural perceptions of ‘outside’ services, alongside exploration of cultural ideas around ‘mental health’ and what ‘help’ consists of.

### Considering cultural differences within the family

Young people alluded to cultural/acculturation differences at home as being at the crux of their presenting difficulties. This finding aligns with research showing variations in acculturation within families may lead to increased conflict (Berry, 2005). Bersani (2014) highlights conflicting family and social expectations as a factor which may contribute to second-generation offending rates. Jeltova et al. (2005) found that intergenerational discrepancies in acculturation between adolescent girls and their parents predicted risky sexualised behaviours. Furthermore, some young people described a sense of isolation from peers, and marginalisation from wider society. Discrimination (de Ruiter and Kaser-Boyd, 2015) and mistrust of the dominant White society (Biafora et al., 1993) have been linked to increased risky behaviours in adolescents.

Similarly to Fox et al.’s (2016) findings, young people described the MST therapist ‘acting as a cultural broker’ by facilitating perspective-taking. Bhugra and De Silva (2000) suggest that cultural-based differences might not always be articulated as the presenting problem, therefore it is the MST therapist’s role to sensitively probe this topic with dual-cultured families. Formulating and sensitively addressing cultural differences, can result in young people feeling understood and being more open about previously ‘taboo’ subjects (Thompson et al., 2007). However, a young person’s participation needs to be considered in the context of cultural differences in communication, the role of family hierarchies, gender norms, and socio-cultural customs around ‘open’ conversations. Walker and Donaldson (2011) suggest that regardless of cultural background, young people may resent their problems being discussed in front of professionals and family members, and can cope by; switching off, opting out, or by agreeing with everything. One-to-one space with a therapist may be particularly valued by minority ethnic youth, given the increased shame associated with their behaviour in their parents’ culture and/or religion (Zhou & Bankston, 1994).

Other young people alluded to a sense that short-term interventions by external services were not sufficient in changing longstanding beliefs held by their parents. MST’s theory of change suggests that for the young person’s behaviour to improve, the therapist helps caregivers to gain resources and skills to be more effective. The efforts of the caregiver is key to change. This study suggests that for some young people, they might see first order change (behaviour) in the short-term, but may be more sceptical around second order change (beliefs and values) in the long-term.

### Understanding the young persons’ unique cultural values/beliefs

Most young people described developing a unique identity; blending elements of their parents’ and peer culture. Schwartz et al. (2006) has called for interventions to promote bicultural identity development in acculturating adolescents, and suggests this may reduce the risk of antisocial behaviours. Initiating conversations around young peoples’ unique cultural identity may help steer the MST therapists’ ideas for positive peer-focused interventions. Additionally, open discussion of the young person’s values/beliefs may alleviate parent concerns around children completely losing their native culture.

### Recognising and reflecting on cultural difference in the therapeutic relationship

In contrast to caregivers (Fox et al., 2016), some young people felt cultural differences between the therapist and family did impact on engagement. Sue and Sue (2012) outlined ways acculturation level might influence the therapeutic encounter; a client who has fully integrated elements of different cultures might feel comfortable working with a therapist of any ethnic background, whereas a client who has devalued the dominant culture may prefer a therapist from his/her own background. Culturally ‘matching’ clients with therapists has been suggested to facilitate culturally-responsive treatment (Sue & Sue, 2012), however research has produced inconclusive results (Maramba & Hall, 2002). Cardemil and Battle (2003) suggest that recognition and discussion of cultural differences may be helpful for engagement.

### Clinical implications

This study highlights several clinical implications and recommendations to MST developers and practitioners working with second-generation minority ethnic young people.

Firstly, cultural/acculturation differences need to be considered when conceptualising referral behaviours. Sam and Berry (2010) suggest that normative developmental issues such as cultural identity, development of self, and family relationships may become complicated by acculturation. MST therapist’s allocating additional time for engaging second-generation young people, and incorporating individual sessions, alongside family work, may be an appropriate way of facilitating whole family engagement and developing a culturally sensitive formulation of presenting difficulties.

Being knowledgeable about culturally-salient issues and how to address these sensitively is fundamental. Ridley (1985) asserted that cross-cultural skill should be on a level of parity with other specialised therapeutic skills; demanding depth of training and supervised experience. Adaptations may be needed in terms of delivery, therapeutic process and inclusion of cultural knowledge to make empirically-supported treatments more culturally appropriate (Whaley and Davis, 2007). These clinical implications can be generalised to other family therapy approaches.

### Limitations

This study excluded minority ethnic young people whose parents were born in the UK, therefore the experiences and views represented in the study constitute a specific group of minority ethnic young people. All caregivers spoke basic English and the majority had lived in the UK over 10 years, therefore the findings might be very different for young people who were first-generation immigrants or whose parents were second-generation or third-generation. Therefore, in this study culture may be confounded with faith, skin colour, migration, being a refugee or seeking asylum. ‘Cultural considerations’ covers a wide area and there are various factors which may have impacted results which have not been discussed in detail or at all.

The sample size was small and relatively diverse, which limits how much can be generalised from the findings. Nevertheless, the sample do share various commonalities; all participants were second-generation, living in London since childhood, and born to parents who were born outside of the European Union. Additionally, this study included families who had dropped-out or not achieved positive outcomes in MST, which added valuable information about the factors which hinder engagement with this specific sub-population. Future research on treatment outcomes for young people with a culturally matched therapist would be useful.

This study attempts to understand the experiences of second-generation young people in MST and expand upon the findings of Fox et al. (2016). The findings suggest that cultural/acculturation differences are important in understanding referral behaviours and the potential complexity of identity development for second-generation young people. There are additional considerations for MST therapists; being knowledgeable and competent in addressing culture, opening conversations about cultural-specific conflicts and the impact of cultural differences on the therapeutic relationship. The findings from this study can inform training for MST teams in order to support the development and learning for working with diverse populations.
